# A *Tequintavirus* bacteriophage SIA3lw isolated from sewage water with antimicrobial potential against antibiotic-resistant *Salmonella* Infantis

**DOI:** 10.1186/s12866-025-04423-4

**Published:** 2025-11-21

**Authors:** Yen-Te Liao, Angela Voelker, Mackenna Chu, Yujie Zhang, Kan-Ju Ho, Leslie A. Harden, Alexandra Salvador, Vivian C.H. Wu

**Affiliations:** Produce Safety and Microbiology Research Unit, U.S. Department of Agriculture, Agricultural Research Service, Western Regional Research Center, Albany, CA 94710 USA

**Keywords:** Lytic bacteriophage, Polyvalent effects, Whole-genome sequencing, *Salmonella* infantis, Antibiotic resistance

## Abstract

**Background:**

*Salmonella enterica* serovar Infantis has consistently contributed to foodborne illness in recent years. Due to frequent antibiotic resistance challenges amid *Salmonella* species, promising antimicrobial alternatives, such as lytic bacteriophages, are considered to improve the efficacy of the antimicrobial interventions currently used in the food industry. Thus, this study aimed to characterize a new phage from sewage water for its antimicrobial potential against antibiotic-resistant *S.* Infantis.

**Methods and results:**

*Salmonella* phage vB_SalS-SIA3lw (or SIA3lw) was isolated from sewage water and subjected to genomic and biological characterization. In vitro antimicrobial activity of SIA3lw was determined against two antibiotic-resistant *S.* Infantis strains. Later, the antibiotic resistance profile of bacteriophage-insensitive mutants (BIMs) was also obtained. The phage has a long, non-contractile tail with a genome size of 116,541 bp and is genomically classified in the *Tequintavirus* genus, sharing a close evolutionary relationship with *Escherichia* phage Bf23. However, SIA3lw shared low nucleotide sequence similarities of receptor binding protein (ORF 22) and putative tail fiber protein (ORF 42) genes—both associated with bacterial host recognition and binding—with that in the Bf23. No genes associated with virulence, antibiotic resistance, and lysogeny were found. For biological traits, SIA3lw has a latent period of 30 min and an estimated burst size of 150 PFU/CFU against *S.* Infantis ATCC BAA-1675. The phage is polyvalent against various *S.* Infantis and two generic *E. coli* strains. Among different multiplicity of infections (MOIs), MOI = 100 was the most effective in reducing antibiotic-resistant *S.* Infantis strains in vitro by more than 4.5 log in 8 h at 25 °C. Despite the occurrence of BIMs, some became sensitive to streptomycin at certain dosages, to which wild-type *Salmonella* strains were resistant. Most importantly, all selected BIMs were susceptible to the infection by a T4-like phage.

**Conclusions:**

Phage SIA3lw has a large burst size and shows strong antimicrobial activities against antibiotic-resistant *S.* Infantis strains. Although BIMs occur after phage treatment, all BIMs are sensitive to a different phage infection. Most of all, some BIMs trade off their resistance to streptomycin at certain dosages for the development of phage resistance. These findings indicate that the newly isolated *Tequintavirus* phage SIA3lw has the antimicrobial potential to mitigate antibiotic-resistant *Salmonella* Infantis.

**Supplementary Information:**

The online version contains supplementary material available at 10.1186/s12866-025-04423-4.

## Introduction

Among more than 2,500 serovars of *Salmonella*, non-typhoid *Salmonella enterica* is one of the top 5 frequently occurring foodborne pathogens in the United States, contributing to 1.35 million cases of *Salmonella*-related illnesses, also known as Salmonellosis, including 26,500 hospitalizations and 420 deaths annually [1, 2]. Non-typhoid *Salmonella enterica* is a zoonotic disease commonly inhabiting the gastrointestinal tract of broilers, and, thus, the animals are the primary contamination source of *Salmonella* [3, 4]. Among these serovars, *S.* Infantis has recently emerged as one of the most common *Salmonella* isolates—primarily from raw meat samples in processing facilities and retail stores—in the United States since 2016 [1, 5] and is one of the leading causes of acute human bacterial gastroenteritis in other parts of the world [6, 7].

A previous study found that *S*. Infantis was the most prevalent serovar of *S. enterica*, contributing to more than 95% of the *Salmonella* isolates from poultry farms and chicken carcasses and 50% of the isolates from human samples [8]. Furthermore, more *S.* Infantis strains isolated from poultry and human origins than any other sources carried the pESI plasmid, among which were resistant to at least one antibiotic, with many being multidrug-resistant [7, 9, 10]. Most importantly, the pESI-like mega-plasmid enables these pathogens to become resistant to disinfectants and to facilitate bacterial adsorption and biofilm-forming ability, which could significantly reduce the effectiveness of the conventional interventions used in the food industry [6]. These findings reveal that the surging contamination of *S.* Infantis poses a new threat to food safety and public health.

With constantly occurring antibiotic resistance among bacterial populations, lytic bacteriophages (phages) embrace a promising antibiotic alternative to mitigate multidrug-resistant bacterial pathogens [11, 12]. Phages are bacterial viruses ubiquitous in the ecosystem [12]. Upon encountering bacterial hosts, the lytic infection starts with phage adsorption to receptors on the bacterial membranes, followed by injecting phage DNA, hijacking bacterial machinery, producing viral DNA and proteins, forming viral particles, and finally causing cell lysis to release the phage progenies [13, 14]. The host-specific killing of a lytic phage primarily relies on binding to specific receptors on the outer bacterial membrane with minimal influence by the antibiotic-resistance characteristics of bacteria; thus, these bacterial predators are promising antimicrobial agents of multidrug-resistant strains [13]. A previous study demonstrated that several *Salmonella*-infecting phages isolated from different water samples showed antimicrobial activities against various *Salmonella* strains, including some with multidrug resistance [15]. Additionally, Sevilla-Navarro et al. isolated multiple lytic phages against *S.* Infantis from broiler farms as alternative disinfection strategies to improve the standard sanitation procedure implemented at the poultry farms [10]. The authors found that phage application significantly reduced *Salmonella* in the environment, such as on the floor, feeder, and drinker. Although there are some studies regarding *S.* Infantis phages, the fundamental (biological and genomic) characterization information is lacking, and the number of phages isolated specific to this serovar is relatively scarce compared to other *Salmonella enterica* serovars, such as *S.* Typhimurium and Enteritidis. Therefore, this study aimed to characterize a newly isolated *Tequintavirus* bacteriophage from sewage water and determine its antimicrobial potential against antibiotic-resistant *S.* Infantis strains.

## Materials and methods

### Bacteriophage isolation

*Salmonella* phage vB_SalS-SIA3lw (or SIA3lw) was isolated from pre-treated sewage water collected at a wastewater treatment plant in northern California. The sewage sample was centrifuged at 8,000 ×*g* for 10 min, and the supernatant was obtained and filtered through a 0.22-µm filter membrane to remove bacterial population and other large-sized microorganisms. Later, an aliquot of 10 mL sample was concentrated through a 100 kDa cut-off Amicon Ultra-15 Centrifugal Filter Unit (Merck Millipore, Billerica, MA, USA). An aliquot of 50 µL concentrated sample was mixed with 100 µL overnight culture of *S.* Infantis ATCC BAA-1675 and 2µL CaCl_2_ and then incubated at room temperature (25 °C) for 5 min before conducting a double-layer plaque assay [16]. After incubating at 37 °C overnight, a single large plaque was picked and resuspended in 1 mL of SM buffer (Thermo Fisher Scientific, Waltham, MA, USA), followed by another round of the double-layer plaque assay (also known as single-plaque purification). At least five runs of the single-plaque purification process were conducted to obtain the final phage.

After purification, the phage was propagated with the overnight bacterial culture of *S.* Infantis ATCC BAA-1675 in 40 mL tryptic soy broth (TSB; Difco, Becton Dickinson, Sparks, MD, USA), supplemented with CaCl_2_ at a final concentration of 10 mM, at 37 °C for 24 h. Subsequently, the phage lysate was centrifuged at 8,000 ×*g* for 5 min before 0.22-µm filtration to remove bacterial debris for further concentration and antimicrobial activity test(s). The phage lysate was further concentrated via a 50 kDa cut-off Amicon Ultra-15 Centrifugal Filter Unit (Merck Millipore, Billerica, MA, USA) and purified via cesium chloride (CsCl) gradient ultracentrifugation to remove bacterial debris, as previously described [17], before downstream analysis, such as phage morphology observation, proteomic analysis, and DNA extraction for whole genome sequencing.

### Bacterial strains

A panel of non-pathogenic *Escherichia coli*, including ATCC 13706, ATCC 15597, and TVS353, Shiga toxin-producing *E. coli* (STEC) strains, including serogroups of O26, O45, O103, O111, O121, O145, and O157, *E. albertii*, and *S. enterica* (Typhimurium, Enteritidis, Montevideo, Newport, Heidelberg, and Infantis) (Table S1) were obtained from the Produce Safety and Microbiology (PSM) Research Unit at the U.S. Department of Agriculture (USDA), Agricultural Research Service (ARS), Western Regional Research Center, Albany, CA, USA. Ten additional *S.* Infantis strains were obtained from the Office of Public Health Science (OPHS) at USDA, Food Safety and Inspection Service (FSIS). These strains were used for the host range, efficiency of plating, and bacterial reduction tests in this study. *S.* Infantis ATCC BAA-1675 was the primary host of phage isolation and was used for phage propagation, the one-step growth curve, and phage quantification of the stability tests. Fresh overnight culture of each selected strain was prepared by inoculating a sterile test tube with 10 mL of TSB with 1 uL loopful of each strain and incubated at 37 °C for 20 h prior to the experiment.

### Genomic analysis

Phage SIA3lw, after CsCl gradient purification, was subjected to DNA extraction using a Norgen phage DNA extraction kit (Thorold, ON, Canada), followed by DNA library preparation via the established protocol [18]. Later, the phage DNA libraries were loaded on a MiSeq Reagent Kit v2 (500-cycle) for the next-generation sequencing using the MiSeq platform (Illumina, San Deigo, CA, USA). Raw sequence reads, 15 million 2 × 250-bp paired-end reads, were subjected to Trimmomatic v.0.38 with the setting of Q30 to remove poor-quality reads. Later, the SPAdes v.3.15.4 [19] was used to conduct *de novo* assembly of the quality reads with the default settings. The resulting contig with N50 contig length of 106,623 bp (also the largest contig) was confirmed as a phage genome via BLASTn before being subjected to the PhageTerm analysis (Galaxy v.1.0.12) to predict the packaging mechanisms and termini of the phage genome [20]. Genome annotation was conducted using the Prokka pipeline Galaxy 1.13 [21], with the default settings, on the re-organized phage sequence obtained from the PhageTerm analysis and further confirmed with the Universal Protein Resource (UniProt) database [22]. tRNAscan-SE (v2.0) was used to predict tRNAs in the phage genome [23]. At the same time, the re-organized genome sequence was subjected to the screening of virulence and antibiotic resistance genes using the VirulenceFinder v2.0 (https://cge.food.dtu.dk/services/VirulenceFinder/; accessed on 03/29/2024) [24] and ResFinder v4.1 web servers (https://cge.food.dtu.dk/services/ResFinder/; accessed on 03/21/2024) [25], respectively, with the default settings. Phage lifecycle, lytic or lysogenic, prediction was determined using the DeepPL [26].

The evolutionary tree based on the whole-genome sequences of SIA3lw and the reference phages belonging to the *Epseptimavirus* and *Tequintavirus* genera, both under the *Demerecviridae* family, was analyzed using the Virus Classification and Tree Building Online Resource (VICTOR) web server [27]. The selected phages from the *Epseptimavirus* and *Tequintavirus* genera were confirmed by the International Committee on Taxonomy of Viruses (ICTV); the myophage Sa157lw was used as an outgroup of the phylogenetic tree. The genome comparison between SIA3lw and its close reference phages was visualized using the pyGenomeViz v.1.3.0. The conservative (core) genes of SIA3lw against the closely related reference phages were analyzed using the CoreGenes3.5 [28]. Phylogenetic analysis of the nucleotide sequences of open reading frame (ORF) 22 and ORF 42 encoding a receptor-binding protein and putative tail fiber protein, respectively, was conducted as previously described [29]. Both ORFs were selected because these were the primary proteins in other reference phages that recognized and adsorbed the receptors on the target bacteria. Additionally, membrane transport proteins, FepA, PhuA, and BtuB, associated with the primary receptors of the reference phages, were confirmed in the previous studies [30, 31]. In brief, the nucleotide sequences were aligned using MAFFT (v 7.490) on Geneious Prime. The phylogenetic trees were performed using the MEGA11 program, with the maximum composite likelihood method and 1,000 bootstrap replications [32].

### Transmission electron microscopy

An aliquot of 5 µL CsCl-purified phage SIA3lw was mixed with 5 µL sterile water for the negative staining preparation and phage morphology observation under the transmission electron microscope (TEM, FEI Tecnai G2) at the University of California, Berkeley EM laboratory using the methods as previously described [29].

### Phage protein analysis

The purified and concentrated phage SIA3lw lysate was subjected to gel electrophoresis, in-gel digestion with trypsin, reverse phase nanoflow high-performance liquid chromatography with tandem mass spectrometry (HPLC-MS-MS), and data analyses as previously described [33]. In brief, the CsCl-purified phage lysate was reduced with 0.5% 2-mercaptoethanol in Laemmli buffer per the manufacturer’s instructions (Bio-Rad, Hercules, CA, USA) and subsequently subjected to SDS-PAGE using a 1D Bio-Rad 12% TGX gel (Bio-Rad, Hercules, CA, USA). The gel was stained using ImperialTM Protein Stain (Thermo Fisher Scientific, Waltham, MA, USA). The gel lane was cut into 47 slices, each of which was placed into a reaction tray and subjected to in-gel digestion with Trypsin (Promega, Madison, WI, USA) using a Digest Pro robot (Intavis, Köln, Germany). Sample digests were analyzed by nanoflow reversed-phase chromatography with an Eksigent NanoLC (Sciex, Framingham, MA, USA) using Picochip columns (New Objectives, Woburn, MA, USA). Data-dependent tandem mass spectra (MS-MS) were obtained in positive ion mode with an Orbitrap Elite mass spectrometer (Thermo Fisher Scientific, Waltham, MA, USA). Mascot software (Matrix Science, Boston, MA, USA) was used to match the MS-MS data to amino acid sequences derived from the nucleotide sequences of phage SIA3lw. Protein identification parameters required a minimum of three identified peptides with a maximum mass error of 10 ppm for the parent ions.

### One-step growth curve

*S.* Infantis ATCC BAA-1675 was used to determine the one-step growth curve of phage SIA3lw using the previous method with subtle modification [29]. In brief, a log-phase bacterial solution was prepared by adding 0.2 mL of fresh overnight culture in TSB into sterile 19.8 mL of TSB for 2-h incubation at 37 °C. Later, phage SIA3lw with MOI of 0.01 was added to the bacterial solution supplemented with CaCl_2_ at 10 mM before 10-minute incubation at 37 °C for phage adsorption. The phage-bacterial mixture was centrifuged at 10,000 × *g* for 5 min at 4 °C to remove the supernatant. The bacterial pellet was washed with 2 mL TSB twice, resuspended with 20 mL fresh TSB, and then 0.3 mL of the resuspension was added to 29.7 mL of fresh TSB. The final solution was incubated at 37 °C to start timing the experiment. Meanwhile, the phage-infected *S.* Infantis cells were determined right before the incubation period (time 0) by mixing 50 µL of the 30-mL final phage-infected bacterial solution (no filtration) with 100 µL of fresh overnight bacterial culture via the double-layer plaque assay. Subsequently, a 1 mL sample was obtained every 5 min from the phage-infected bacterial solution at 37 °C and filtered through a 0.22-µm filter membrane. The titers of phage SIA3lw at each time point were determined using the double-layer plaque assay described above. The experiment was conducted in three replications to estimate the latent period—the time required from phage infection to bacterial cell lysis to detect phage progenies—and the burst size of phage SIA3lw.

### Phage stability (pH, temperature, and storage)

Phage SIA3lw was subjected to the pH stability test, from pH 3 to pH 12, using the method previously described [34]. In brief, phage SIA3lw (~ 10 log PFU/mL) with 100 µL was added into 4.9 mL of SM buffer to reach the final pH values of 3, 4, 5, 7, 10, 11, and 12. Samples were incubated at 30 °C for 24 h before enumerating viable virions using the double-layer plaque assay. Regarding the thermal stability test, SIA3lw solution was prepared in SM buffer to reach approximately 8 log PFU/mL before dispensing 0.5 mL of the phage solution in several sterile microcentrifuge tubes. The phage was placed in different water baths set to 30 °C (control), 40 °C, 50 °C, 60 °C, and 65 °C for 24-h thermal treatment. The temperatures used in this study covered possible pre-harvest conditions and various post-harvest food processing settings. Phage concentrations were then determined using the double-layer plaque assay. For the storage test, phage SIA3lw with 8 log PFU/mL was prepared in SM buffer before aliquoting 0.5 mL phage per tube in multiple sterile microcentrifuge tubes and then stored at 4 °C for 128 days. All experiments were conducted in 3 replications.

### Host range and efficiency of plating

The host range test of SIA3lw against non-pathogenic *E. coli*, seven primary STEC serogroups, *E. albertii*, and various *S. enterica* serovars, primarily Infantis, strains was determined using a spot test assay as previously described [34]. As a result, the susceptible strains were subjected to the efficiency of plating (EOP) assay [33] to determine productive infection of phage SIA3lw by measuring the phage progenies produced from the tested strains versus those produced from the primary strain of *S.* Infantis ATCC BAA-1675. Briefly, a fresh bacterial culture of the selected strains was prepared in TSB at 37 °C overnight and used for phage SIA3lw quantification with four successive dilutions of the phage (10^−3^ to 10^−7^) via the double-layer plaque assay. The plates were incubated at 37 °C for 20 h for plaque quantification and EOP calculation. The experiment was conducted in three replications. Generally, a high phage-producing efficiency had an EOP of 0.5 or more; a medium-producing efficiency had an EOP above 0.1 but below 0.5; a low-producing efficiency had an EOP between 0.001 and 0.1; inefficient phage production was any value lower than 0.001.

### Lysis from without

The lysis from without (LO) was conducted for phage SIA3lw at different MOIs against *S.* Infantis strains (FSIS9799 and FSIS4897) using a spectrophotometer. The bacterial culture of each strain was prepared in 10 mL TSB and incubated at 37 °C for 20 h. The next day, the overnight culture was diluted to 5 log CFU/mL in TSB as the final concentration, and then 180 µL of the bacterial culture was dispensed to three wells per MOI for each strain using a 96-well plate. Subsequently, an aliquot of 20 µL phage solution at MOIs of 10 and 100 was added to the designated wells. The control group contained the bacterial culture with 20 µL SM buffer without phage. The optical density at 600 nm (OD_600_) was recorded every 5 min for 20 min at 25 °C.

### Antimicrobial activity against antibiotic-resistant S. Infantis in LB broth

Two *S.* Infantis strains (FSIS9799 and FSIS4897) with different antibiotic resistance profiles were separately used to evaluate the antimicrobial activity of SIA3lw following the method previously described with subtle modifications [35]. In brief, each bacterial culture was prepared in 10 mL TSB at 37 °C for 20 h and then used to prep the final bacterial concentration in lysogeny broth (LB; Invitrogen, Carlsbad, CA, USA) at approximately 5 log CFU/mL before dispensing 20 mL of the bacterial solution in four sterile 50-mL conical tubes. At the same time, phage SIA3lw, diluted in SM buffer, was added to each tube to reach MOIs of 1, 10, and 100, respectively. For the control, SM buffer, with the same volume as the phage used in the treatment, was also added to a 20-mL bacterial solution. Control and treatment groups were incubated at 25 °C for 24 h, and the bacterial levels were confirmed via plating on xylose lysine deoxycholate (XLD; Hardy Diagnostics, Santa Maria, CA, USA) overlayered thin tryptic soy agar (TSA), also known as Thin Agar Layer Method (TAL) [36], at the time points of 0, 2, 4, 6, 8, and 24 h. The plates were incubated at 37 °C overnight for bacterial quantification. The experiment was conducted in three replications.

### Bacteriophage-insensitive mutant screening

A total of 90 colonies, with 30 per MOI, was obtained for each phage-treated *S.* Infantis (FSIS9799 or FSIS4897) from XLD TAL plates at the 24-h time point to screen for bacteriophage-insensitive mutants (BIMs) using the previous method with modification [29]. In brief, the colonies were picked from the plates of 24-h phage treatment to prepare overnight cultures in TSB at 37 °C. The overnight cultures of phage-untreated *S.* Infantis (FSIS9799 or FSIS4897) strains were used as control. An aliquot of 0.1 mL TSB was loaded in each well of a 96-well plate to mix with 0.1 mL of each overnight bacterial culture (two wells per bacterial strain). Later, an aliquot of 0.01 mL of phage SIA3lw at 8 log PFU/mL was added to one well, and 0.01 mL of SM buffer was added to the other. After overnight incubation at 37 °C, the 96-well plate was subjected to OD_600_ reading using a spectrophotometer. The bacterial strain displayed sensitivity to phage infection if the OD_600_ value of the well with phage was lower than that with SM buffer (control group). If the OD_600_ values were within the range between the wells with and without the phage, the bacterial culture, from the well of SM buffer, was then streaked on a TSA plate and incubated at 37 °C overnight (repeated 3 times). Later, the overnight cultures were prepared in TSB from the 3rd TSA plate for the phage susceptibility test using the spot test assay described in the host range test section. The bacterial strains that did not form lysis zones were considered presumptive BIMs. The susceptibility of these presumptive BIMs to a different lytic phage was determined using phage vB_EcoM-Sa45lw (or Sa45lw) previously isolated and characterized in our lab [35].

### Antibiotic resistance profile testing

Two selected *S.* Infantis strains (FSIS9799 and FSIS4897) and several BIMs obtained after 24-h SIA3lw treatment were subjected to the antimicrobial resistance test using the agar disc diffusion method based on the guidelines of the Clinical & Laboratory Standards Institute (CLSI; CLSI M02, Performance Standards for Antimicrobial Disk Susceptibility Tests, 14th Edition). The bacterial cultures were prepared in 10-mL TSB at 37 °C for 18 h. The next day, an aliquot of 0.2 mL of overnight culture was plated onto Mueller-Hinton Agar (MHA, Oxoid, Hampshire, United Kingdom) plates and air-dried under the hood for 10 min. Subsequently, the antibiotic discs of streptomycin 10 µg (S), cefoperazone 75 µg (CFP), kanamycin 30 µg (K), ampicillin 10 µg (AMP), ceftriaxone 30 µg (CRO), chloramphenicol 30 µg (C), trimethoprim-sulfamethoxazole 25 µg (SXT), and cefazolin 30 µg (KZ) (Oxoid, Hampshire, United Kingdom) were placed separately onto the surface of MHA plates with bacterial layer, followed by incubation at 37 °C overnight. The zone diameter breakpoints were recorded in centimeters according to CLSI standard guidelines.

### Statistical analysis

Experiments were performed with three individual repetitions. Bacterial colony counts and phage titers were calculated as CFU/mL or PFU/mL and logarithmically transformed for statistical analysis. One-way analysis of variance (ANOVA) with statistical significance at a 5% level was used to evaluate the effects of pH, temperature, and storage tests on the recovery of phage titers. 

## Results

### Genomic and comparative analyses of SIA3lw

**Fig. 1:**
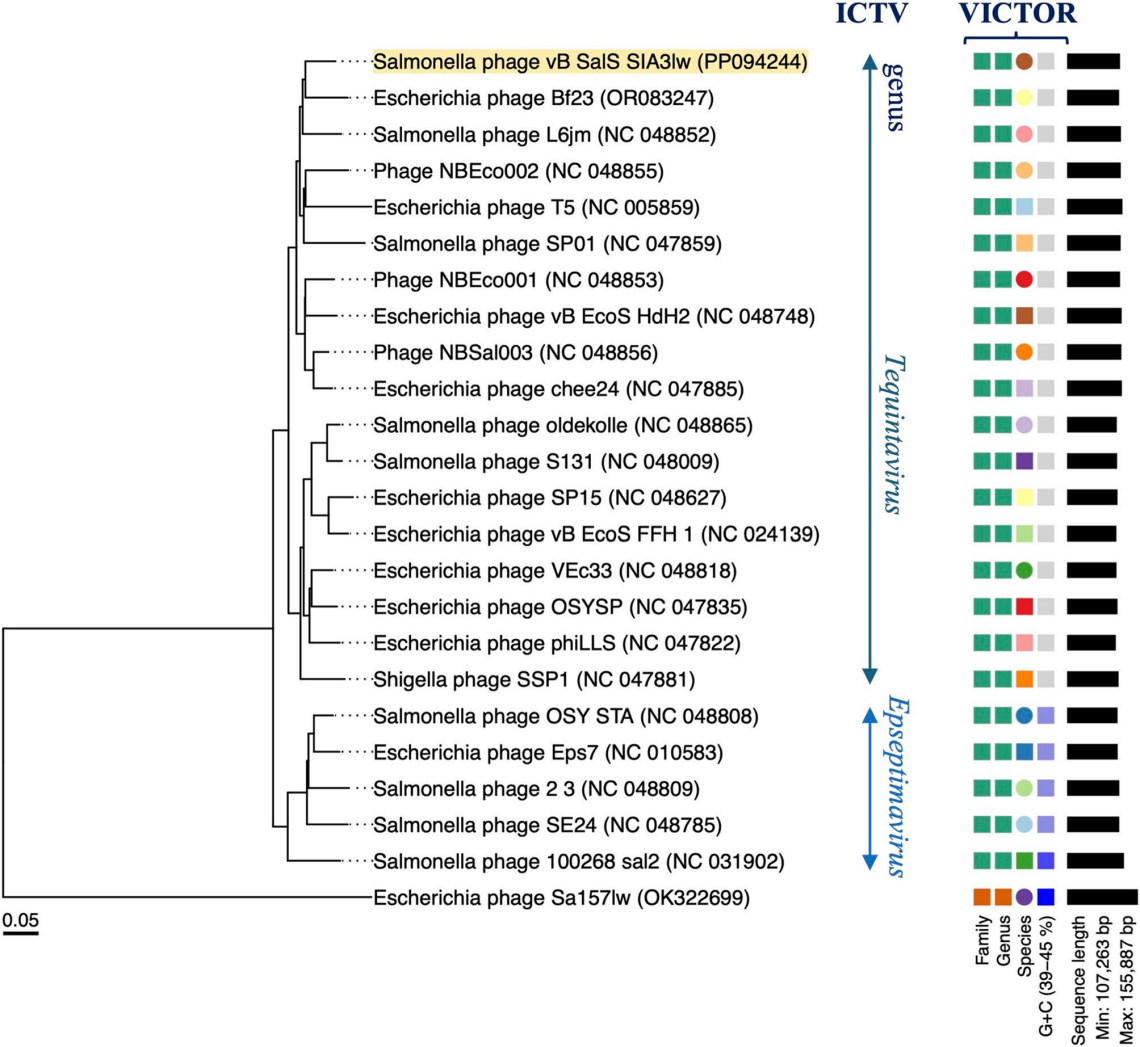
The whole-genome phylogenetic analysis of SIA3lw and reference phages. The analysis is conducted on SIA3lw and the closely related reference phages belonging to the *Epseptimavirus* and *Tequintavirus* genera under the *Demerecviridae* family, confirmed by ICTV, at the nucleic acid level using VICTOR (formula d0). Phage Sa157lw from a different genus is used as an outgroup. Annotations, including family, genus, and species cluster predicted by VICTOR, genomic G + C content, and sequence length, are given on the right-hand side of the tree. The accession number of each phage is provided in parentheses

**Fig. 2:**
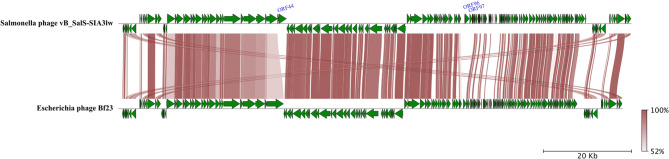
Genome comparison of the whole genome of SIA3lw and its close-related *Escherichia* phage Bf23 using pyGenomeViz. Whole genome maps are presented as green arrows, indicating the order of annotated ORFs from left to right along the phage genomes. The red-scale shaded area indicates the difference in sequence similarity. Three ORFs—ORF 44, ORF 96, and ORF 97 encoding putative peptidase S74 domain-containing protein, band 7 domain-containing protein, and DNA primase, respectively—from SIA3lw are not found on the Bf23 genome. The sequence direction is adjusted prior to the analysis

SIA3lw contained a double-stranded DNA phage with a genome size of 116,541 bp and an average GC content of 39.1%. The phylogenetic analysis based on whole-genome sequences via the VICTOR analysis demonstrated SIA3lw sharing a close evolutionary relationship with *Escherichia* phage Bf23 at the nucleic acid level among the selected reference phages belonging to the *Tequintavirus* genus under the *Demerecviridae* family (Fig. 1). In addition, the pairwise comparison results indicated that SIA3lw shared an average nucleotide identity calculated based on BLAST+ (ANIb) of 90.68% over 74.15% query coverage and ANIb of 90.69% over 79.76% query coverage to *Tequintavirus* phages T5 and Bf23, respectively, calculated by JSpeciesWS web server [37]. As a result, SIA3lw also belonged to the *Tequintavirus* genus according to the ICTV taxonomy guideline [38]. Furthermore, the comparative analysis revealed that SIA3lw contained the primary regions not found in the Bf23 genome, including ORF 44, ORF 96, and ORF 97 encoding putative peptidase S74 domain-containing protein, band 7 domain-containing protein, and DNA primase, respectively (Fig. 2). Additionally, ORF 22 (coding for receptor binding protein), ORF 34 (coding for tail fiber protein), ORF 42 (coding for putative tail fiber), and ORF 94 (coding for recombination related exonuclease) in SIA3lw shared low nucleotide sequence similarities with that in Bf23 genome (Fig. 2).

**Fig. 3:**
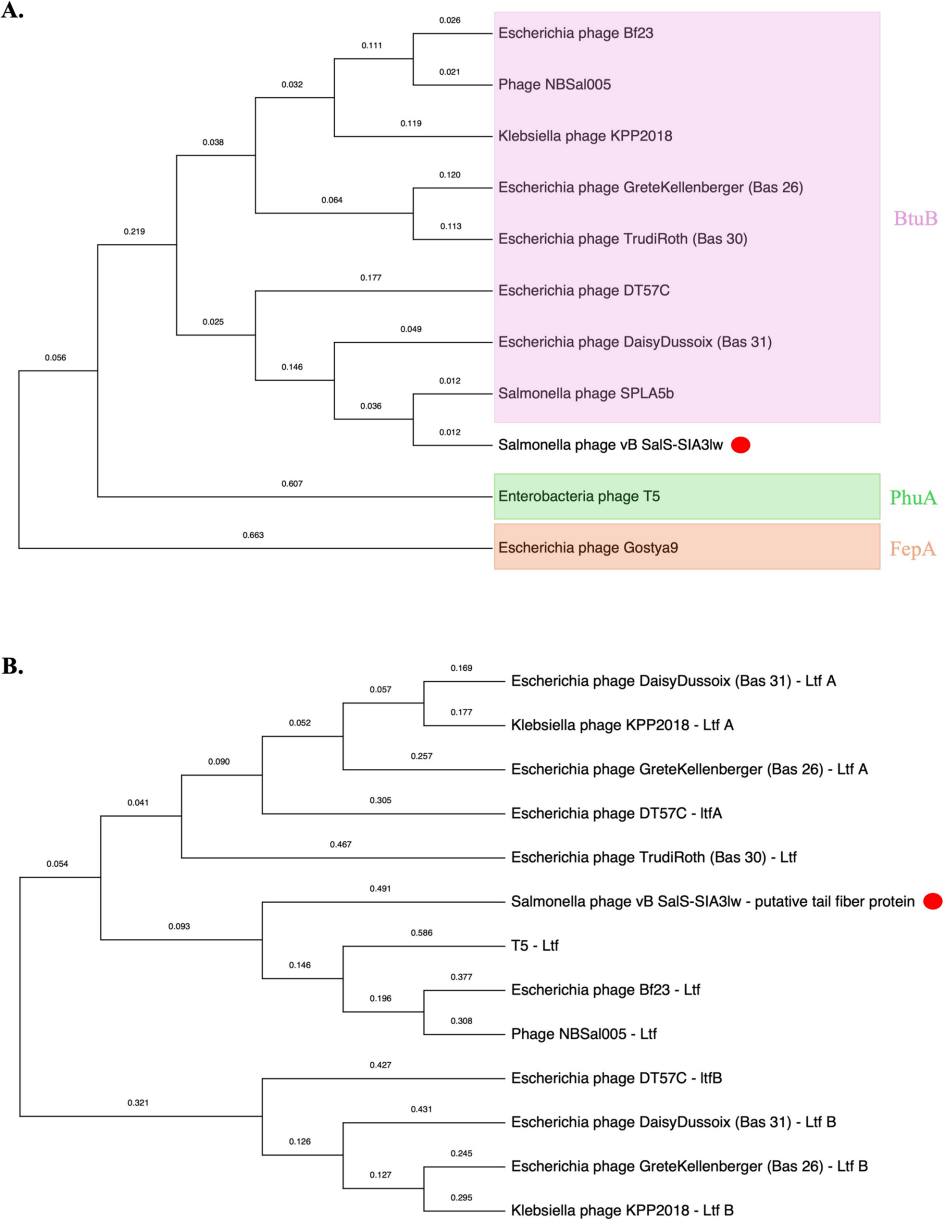
Phylogenetic tree of phage proteins associated with bacterial host recognition. The analysis of phage SIA3lw and the reference phages belonging to the *Tequintavirus* genus based on the Clustal Omega alignment of the nucleotide sequences of the (**A**) receptor-binding protein (ORF 22) and (**B**) putative tail fiber (ORF 42). Membrane transport proteins, such as FepA, PhuA, and BtuB, serve as primary receptor proteins for the reference phages

Phage SIA3lw encoded 191 ORFs, of which 89 were annotated with known functions and 23 tRNAs (Table S2), with long terminal repeats (~ 9.9 k bp) similar to phage T5 [39]. Among the known ORFs, the predicted functions were associated with phage structural proteins (capsid protein and tail), bacterial host receptor binding proteins, host lysis (holin, endolysin, and spanin), phage DNA replication, and host cell regulation and metabolism (Table S2). In silico analysis showed that SIA3lw did not contain ORFs associated with lysogeny, bacterial virulence, and antibiotic resistance. Additionally, the results of DeepPL predicted that SIA3lw was a lytic phage. Based on the phylogenetic analysis of receptor binding protein, phage SIA3lw was closely associated with *Salmonella* phage SPLA5BH and in the group of the phages capable of targeting protein BtuB, including phage Bf23 (Fig. 3A). As for the putative tail fiber, similar to L-shape tail fiber (Ltf), facilitating host recognition and binding among T5-like phages, SIA3lw shared a high nucleotide sequence similarity with *Escherichia* phage Bas30, which did not contain LtfA and LtfB (Fig. 3B). Phage SIA3lw was at a different clade from phage Bf23, aligning with the results of the low sequence similarity of the tail fiber protein (likely associated with host binding) between phages SIA3lw and Bf23, previously mentioned in (Fig. 2).

### Morphology

**Fig. 4:**
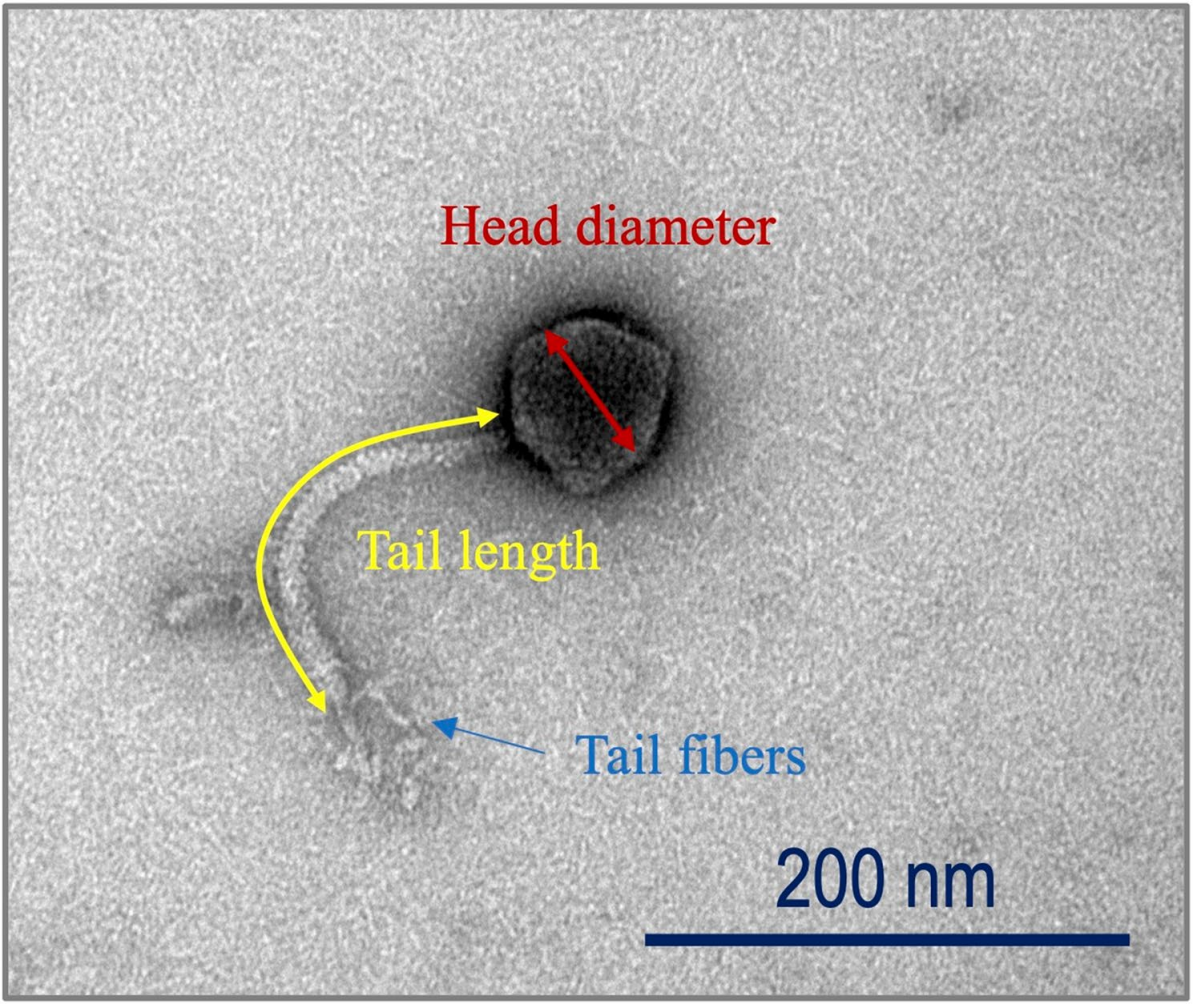
Morphology of phage SIA3lw. Transmission electron microscopy image of SIA3lw with an icosahedral capsid (76 ± 4 nm in diameter), a non-contractile tail (192 ± 8 nm in length), and tail fibers

Phage SIA3lw has a siphovirus morphology that contains an icosahedral head with 76 ± 4 nm in diameter, a long non-contractile tail of 192 ± 8 nm, and tail fibers at the tip of the tail structure (Fig. 4).

### Phage protein analysis

**Fig. 5:**
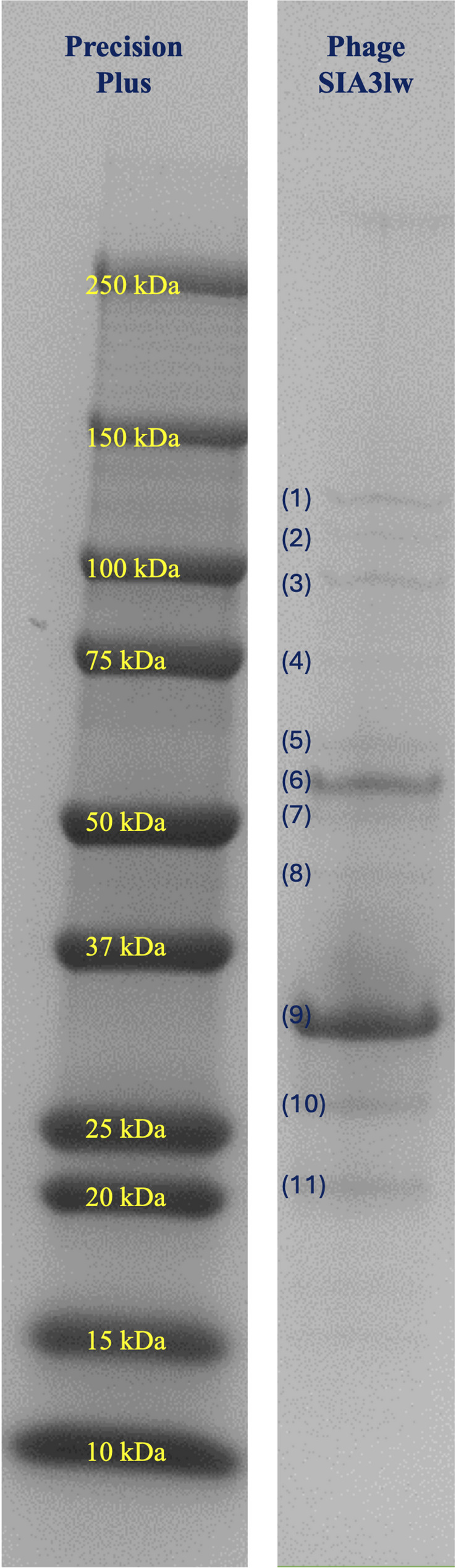
The phage SIA3lw proteins stained with Coomassie Brilliant Blue R-250 and visualized on a 12% SDS-PAGE gel. (1) = tape measure protein; (2) = tail protein; (3) = putative tail fiber protein; (4) = tail protein; (5) = putative peptidase S74 domain; (6) = tail tube protein; (7) = portal connector protein; (8) = ribonucleoside-diphosphate reductase; (9) = major capsid protein; (10) = D11 protein; (11) = head completion protein. Refer to Table 1 for the corresponding ORFs and the rest of the protein information

Fifteen bands related to phage SIA3lw proteins were separated through sodium dodecyl sulfate-polyacrylamide gel (SDS-PAGE), with molecular weights ranging from approximately 19.1 to 132.3 kDa (Fig. 5). The identified gel bands, labeled with 1 to 11, included various structural phage proteins, such as tape measure proteins, tail proteins, putative tail fiber proteins, putative peptidase S74 domain, tail tube proteins, portal connector proteins, major capsid protein, and head completion protein, etc., with the coverage of amino acid sequences ranging from 46 to 90% by mass spectrometry (Table 1). Additionally, phage DNA replication-associated proteins, such as ribonucleoside-diphosphate reductase and D11, were identified at gel bands 8 and 10, respectively. All identified protein bands by mass spectrometry matched the genome annotation results of SIA3lw (Table 1).

**Table 1:** Phage protein analysis of SIA3lw, identified by high-performance liquid chromatography with tandem mass spectrometry (HPLC-MS-MS)

Gel band	ORF	Predicted function	MW kDa	No. of peptides	Sequence coverage
1	SIA3lw_00037	tape measure protein	132.33	69	62%
2	SIA3lw_00039	tail protein	106.95	35	46%
3	SIA3lw_00042	putative tail fiber protein	82.16	38	63%
4	SIA3lw_00040	tail protein	74.84	23	48%
5	SIA3lw_00044	putative peptidase S74 domain	64.30	20	46%
6	SIA3lw_00033	tail tube protein	50.61	19	67%
7	SIA3lw_00026	portal connector protein	45.36	24	65%
8	SIA3lw_00078	ribonucleoside-diphosphate reductase	43.59	14	45%
9	SIA3lw_00029	major capsid protein	50.76	33	57%
10	SIA3lw_00050	D11 protein	28.90	14	56%
11	SIA3lw_00030	head completion protein	19.14	11	90%

### One-step growth curve

**Fig. 6:**
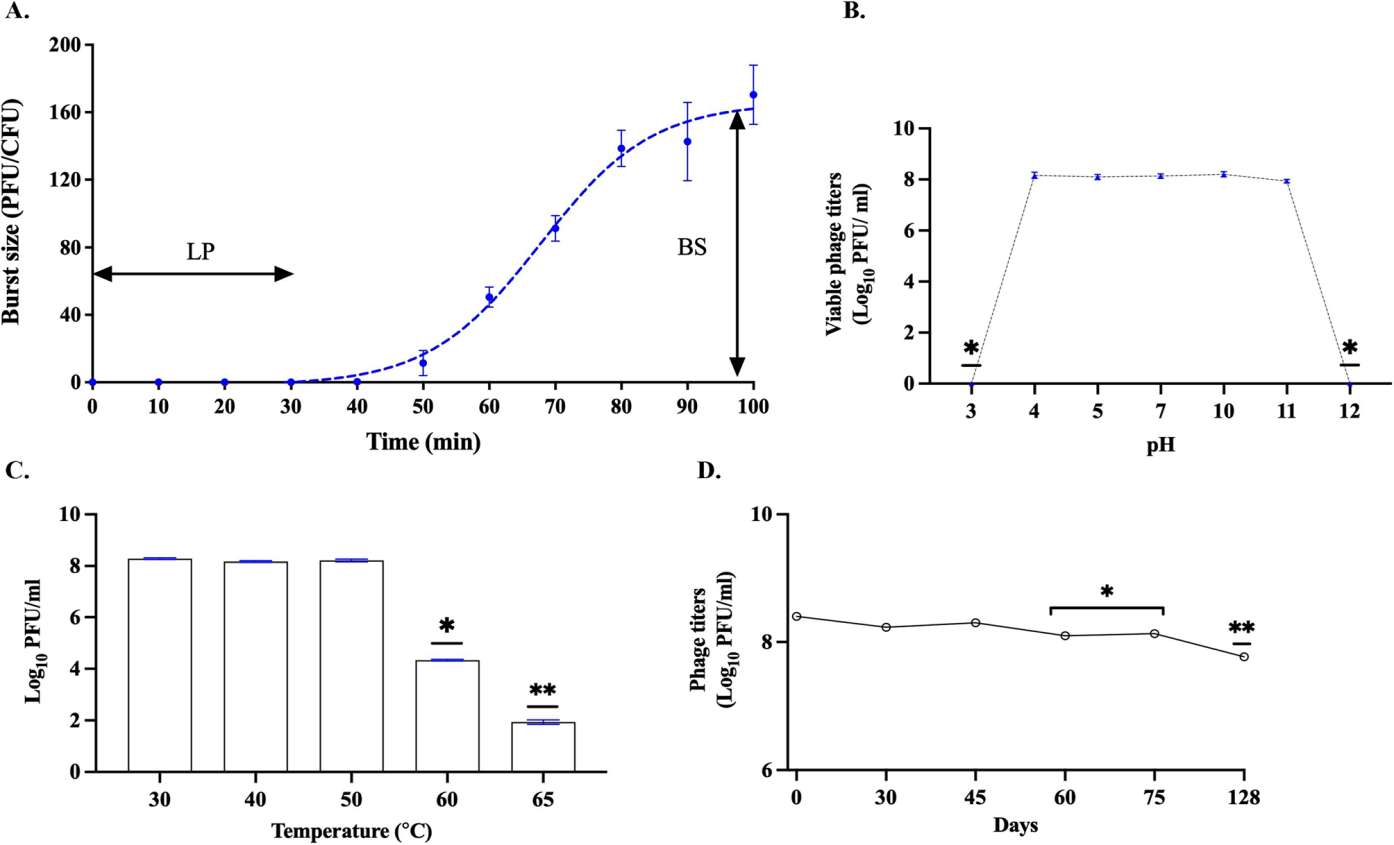
Biological characterization of phage SIA3lw. **A** The one-step growth curve on *S.* Infantis BAA-1675 with a latent period (LP) of 30 min and an average burst size (BS) of 150 PFU per infected cell. **B** The pH stability test for 24 h at 30 °C. **C** The temperature stability test for 24 h. **D** The refrigerated storage test at 4 °C for 128 days. For stability tests, the means of phage titers that contain different numbers of asterisks differ (*P < 0.05*). The error bars show the standard error of the mean (SEM)

The growth factor of one-step growth curve showed that phage SIA3lw had a latent period of 30 min with an estimated burst size of 150 PFU/CFU after MOI of 0.01 was used to infect *S.* Infantis ATCC BAA-1675 in TSB at 37 °C (Fig. 6A). The result showed that a complete lytic cycle for SIA3lw was about 100 min.

### Phage temperature, storage, and pH stability

For the pH stability test, phage SIA3lw maintained similar virion levels from pH 4 to pH 11 for 24 h at 30 °C. (Fig. 6B). However, the phage was reduced below detected levels at pH 3 and pH 12, respectively (Fig. 6B). Regarding the thermal stability test, SIA3lw was stable from 30 °C to 50 °C but significantly decreased at 60 and 65 °C by over 3.5 and 6 log, respectively (Fig. 6C). Additionally, the phage remained at similar levels at 4 °C for 45 days before the first titers decreased on the 60th day (*P < 0.05*) and further dropped on day 128 (*P < 0.05*) of the storage test (Fig. 6D).

### Host range and productive infection of SIA3lw

Among different *S. enterica* serovars, phage SIA3lw showed lytic infection against various *S.* Infantis strains besides *S.* Infantis FSIS9851 (Table 2). In addition to the bacterial host for the phage isolation, phage SIA3lw showed high production efficiency after infecting most *S.* Infantis strains except for *S.* Infantis RM2480 (inefficiency) and *S.* Infantis FSIS4921 (low production efficiency). Furthermore, the phage demonstrated high productive infection against generic *E. coli* ATCC 13706 and ATCC 15597, with EOP of 6.9 and 5.4, respectively, but did not show lytic effects against any pathogenic *E. coli* strains (Table 2). Thus, SIA3lw was a polyvalent phage targeting both *Salmonella* and *E. coli* species.

**Table 2:** Host range test and EOP of phages SIA3lw against various *Salmonella* and *E*. *coli* strains

Strains	Strain Ref. No.	EOP
*Salmonella*	*Salmonella* Infantis (ATCC BAA-1675)	H
	*Salmonella* Infantis (RM2480)	< 0.001
	*Salmonella* Infantis (RM2481)	1.2
	*Salmonella* Infantis (RM19091)	1.4
	*Salmonella* Infantis (RM19096)	2.1
	*Salmonella* Infantis (FSIS9799)	2
	*Salmonella* Infantis (FSIS9916)	1.7
	*Salmonella* Infantis (FSIS4897)	0.9
	*Salmonella* Infantis (FSIS4900)	2.1
	*Salmonella* Infantis (FSIS4921)	0.06
	*Salmonella* Infantis (FSIS9851)	R
	*Salmonella* Infantis (FSIS9861)	2.9
	*Salmonella* Infantis (FSIS7821)	2.8
	*Salmonella* Infantis (FSIS5221)	1.9
	*Salmonella* Infantis (FSIS7823)	3.2
	*Salmonella* Typhimurium 3142	R
	*Salmonella* Typhimurium 14028	R
	*Salmonella* Montevideo S1	R
	*Salmonella* Newport H1073	R
	*Salmonella* Heidelberg 45955	R
	*Salmonella* Enteritidis PT-30	R
Generic *E. coli*	ATCC 13706	6.9
	ATCC 15597	5.4
	TVS353	R
STEC	O26 (SJ2), O45 (RM10729), O103 (RM10744), O111 (RM11765), O121 (RM8082), O145 (RM13514), and *E. coli* O157:H7 (ATCC 35150 & ATCC 43888)	R
*E. albertii*	RM9973, RM9974, RM15113, RM15115, and RM10705	R

^*^EOP was calculated by the ratio of phage titer on a test bacterium versus the primary bacterial host. High production efficiency is EOP ≥ 0.5, medium production efficiency is 0.5 > EOP ≥ 0.1, low production efficiency is 0.1 > EOP > 0.001, and the inefficiency of phage production is EOP ≤ 0.001

H means the bacterial host used for the phage isolation and propagation

R means the bacterial strain resistant to the phage infection without any bacterial lysis

### Antimicrobial activity against antibiotic-resistant S. Infantis in LB broth

**Fig. 7:**
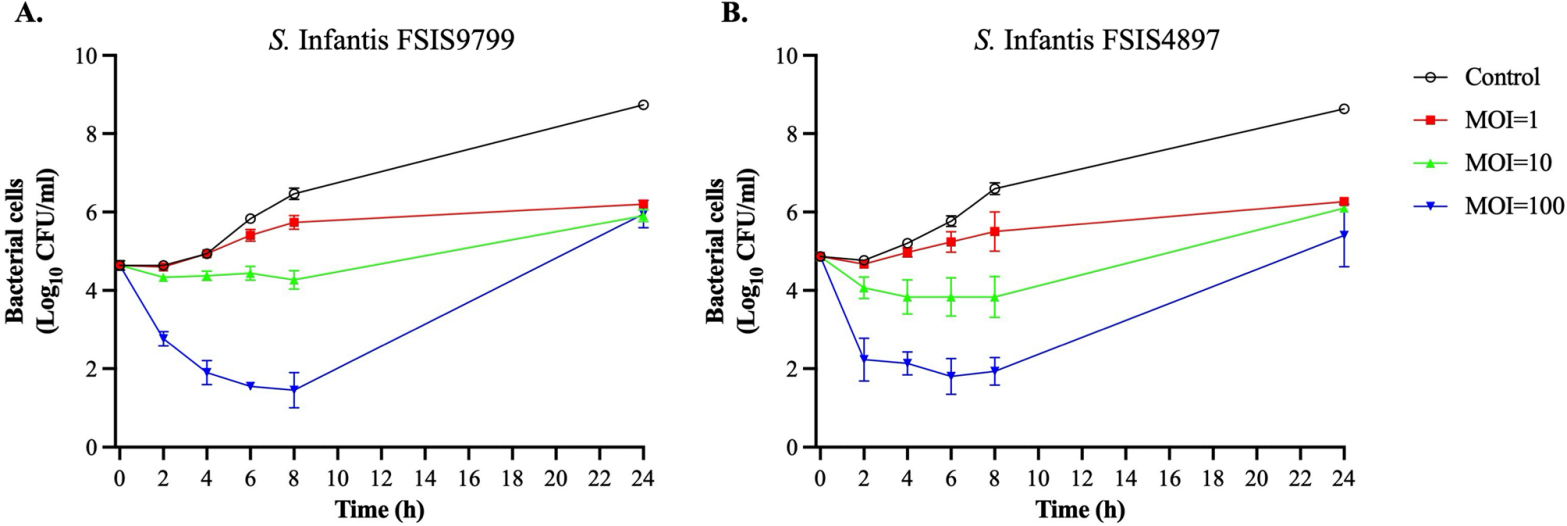
In vitro antimicrobial activity of phage SIA3lw against antibiotic-resistant *S.* Infantis. The phage with MOIs of 1, 10, and 100 is used to treat antibiotic-resistant (**A**) *S.* Infantis FSIS9799 and (**B**) *S.* Infantis FSIS4897 strains in lysogeny broth at 25 °C for 24 h. The control group only contains bacterial culture. The error bars present the SEM

**Fig. 8:**
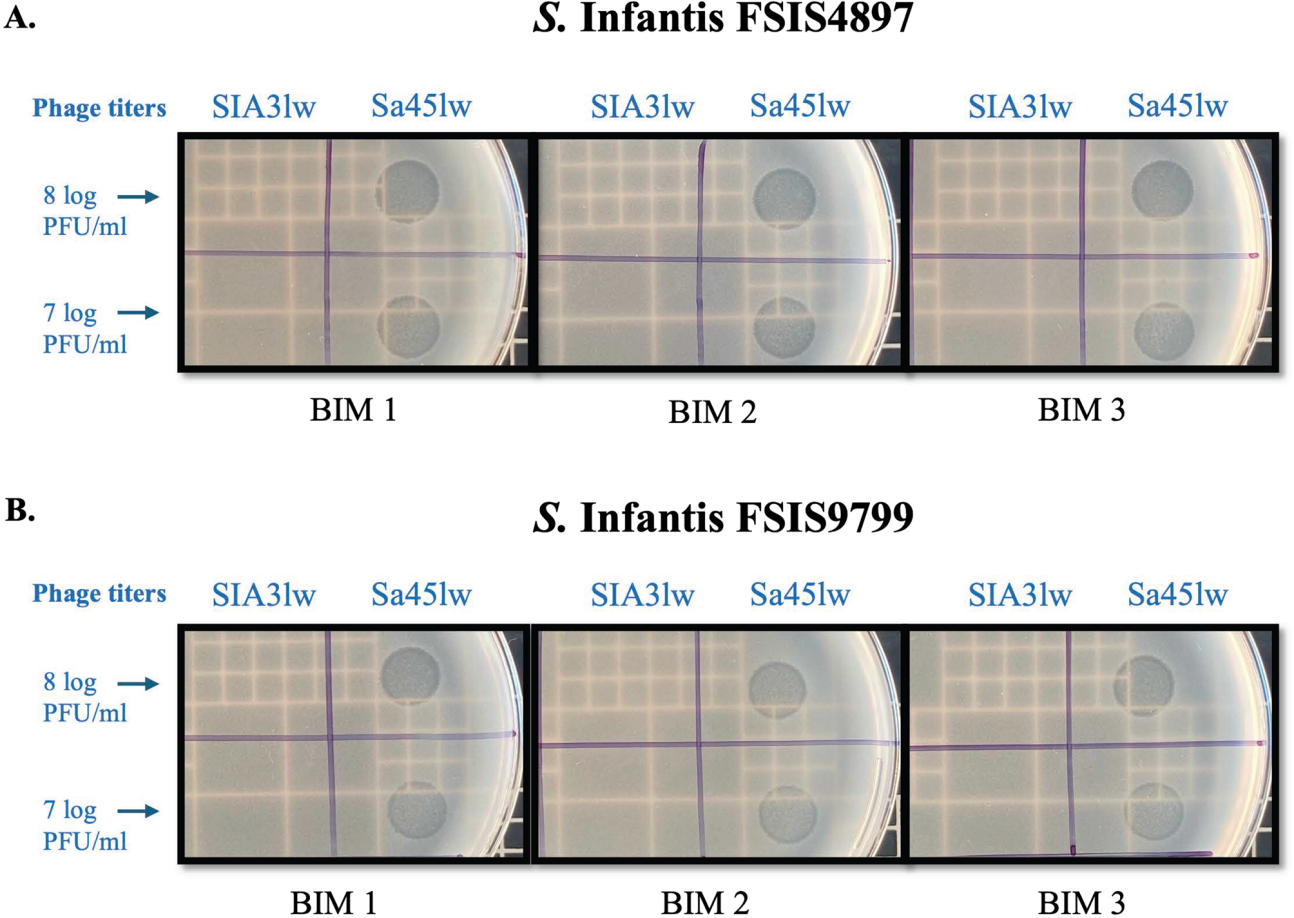
Susceptibility of BIMs to different phage infections. Spot test assay for phages SIA3lw and Sa45lw, with the titers of 7 and 8 log PFU/mL, against the BIMs obtained from SIA3lw-treated (**A**) *S.* Infantis FSIS4897 and (**B**) *S.* Infantis FSIS9799. The presence of circular lysis zones indicates bacterial lysis by the phage infection

The in vitro antimicrobial activity of SIA3lw, with different MOIs, against antibiotic-resistant *S.* Infantis (FSIS9799 and FSIS4897) was evaluated in LB broth at 25 °C (Fig. 7). The LO screening demonstrated that no effect of LO from MOI = 10 and 100 was detected, suggesting the lytic infection of SIA3lw was the primary effect in the bacterial reduction. The results showed that the antimicrobial activities increased as the phage treatment with the higher MOIs was used, with the MOI of 100 being most effective in bacterial reduction of *S.* Infantis (FSIS9799 and FSIS4897). At MOI = 1, the bacterial reduction of *S.* Infantis FSIS9799 and FSIS4897 was not distinct until 6-h and 4-h of time points, respectively. Regarding MOI = 10, *S.* Infantis FSIS9799 was reduced after the 2-h treatment but maintained at a similar level until 8 h. Although the bacterial level of *S.* Infantis FSIS4897 dropped after 2 h, the bacterial reduction was higher than that of *S.* Infantis FSIS9799 until the 8-h time point. For MOI = 100, the bacterial populations were reduced continuously until the 8-h time point for both *S.* Infantis (FSIS9799 and FSIS4897), with the highest reduction of 5.0 and 4.7 log, respectively, compared to the control groups (Fig. 7A and B). After 24-h treatment, *S.* Infantis FSIS9799 treated with SIA3lw at both MOIs = 10 and 100 grew back to similar levels as MOI = 1; however, the bacterial levels of the treatment groups were lower than the control by approximately 2 log (Fig. 7A). For *S.* Infantis FSIS4897, despite a similar phenomenon observed at 24-h timepoint, the bacterial levels from the MOI = 100 were lower than the other treatment groups (MOIs = 1 and 10) (Fig. 7B). Moreover, the BIM screening results showed that 30% and 46.7% of the *S.* Infantis FSIS9799 and FSIS4897 colonies (*n* = 90), respectively, obtained from the phage treatment were presumptive BIMs, regardless of MOI use; the treatment with the higher MOI might increase the occurrence of *S.* Infantis presumptive BIMs for both bacterial strains used in this study (Table S3). Interestingly, the results revealed that few presumptive BIMs, regardless of *Salmonella* strains, became sensitive to streptomycin at certain dosages, but only one *S.* Infantis FSIS9799 presumptive BIM became sensitive to streptomycin and chloramphenicol, both at certain dosages (Table 3). In addition, all the selected presumptive BIMs from the treatment of SIA3lw showed susceptibility to another lytic T4-like phage, Sa45lw, previously isolated in our lab (Fig. 8). Overall, SIA3lw demonstrated proficient in vitro antimicrobial activities against both *S.* Infantis strains in 8 h when MOI of 100 was used. Using a phage cocktail will be a solution to minimize the development of BIMs during phage treatment.

**Table 3:** Antibiotic susceptibility test of *S.* Infantis (FSIS9799 and FSIS4897) and their presumptive BIMs obtained after 24-h phage treatment

Strains	Antibiotics						
	Streptomycin	Cefoperazone	Kanamycin	Ampicillin	Ceftriaxone	Chloramphenicol	Cefazolin
*Wild-type S. Infantis FSIS9799*	R	R	R	R	R	R	R
*BIM 1_FSIS9799*	I	R	R	R	R	I	R
*BIM 2_FSIS9799*	R	R	R	R	R	R	R
*BIM 3_FSIS9799*	I	R	R	R	R	R	R
*BIM 4_FSIS9799*	R	R	R	R	R	R	R
*BIM 5_FSIS9799*	R	R	R	R	R	R	R
*BIM 6_FSIS9799*	I	R	R	R	R	R	R
*BIM 7_FSIS9799*	I	R	R	R	R	R	R
*Wilde-type S. Infantis FSIS4897*	R	S	S	I	S	S	S
*BIM 1_FSIS4897*	R	S	S	I	S	S	S
*BIM 2_FSIS4897*	R	S	S	I	S	S	S
*BIM 3_FSIS4897*	R	S	S	I	S	S	S
*BIM 4_FSIS4897*	R	S	S	I	S	S	S
*BIM 5_FSIS4897*	R	S	S	I	S	S	S
*BIM 6_FSIS4897*	R	S	S	I	S	S	S
*BIM 7_FSIS4897*	I	S	S	I	S	S	S

Resistant (R) means the inhibition zone with the diameter size falling under the resistant category based on the guidelines

Intermediate (I) means the bacterium is sensitive to the drug at certain dosages (buffer zone between susceptible and resistant)

Susceptible (S) means the bacterium is sensitive to the drug

## Discussion

*Salmonella* serovar Infantis has emerged as a new bacterial pathogen among numerous foodborne outbreaks associated with poultry in recent years. This pathogen not only shows high tolerance to many disinfection methods, such as heat, osmotic pressure, or acid [40], but also carries antibiotic-resistant features [40] and an increased biofilm-forming ability compared to other *Salmonella* serovars [41]. These factors enable the pathogen to evade the common antimicrobial interventions used in the food industry and subsequently cause human infection after consuming the contaminated products. For this reason, developing a novel and effective antimicrobial agent, such as lytic phages, is necessary to improve the insufficiency of conventional antimicrobial interventions [42]. Moreover, the U.S. Food and Drug Administration (FDA) has approved various phage products as Generally Recognized As Safe (GRAS), which sheds promising light on the use of phage applications to improve food safety [43, 44].

The current study emphasized the characterization of a newly isolated phage and its antimicrobial potential against antibiotic-resistant *S.* Infantis strains. Phage SIA3lw was phylogenetically similar to phage Bf23 at nucleotide sequence among 24 reference phages used in this study, sharing more than 70% nucleotide identity over the full genome length of Bf23, thus belonging to the *Tequintavirus* genus based on the ICTV taxonomy guideline [38]. Furthermore, SIA3lw harbored a similar number of tRNAs and phage termini (long terminal repeats) as most *Tequintavirus* (T5-like) phages. Although SIA3lw can infect *Salmonella* and *E. coli*, similar to most T5-like phages, such as Bf23 and T5 [45, 46], the difference between phages SIA3lw and Bf23 falls on the sequence regions coding for the proteins associated with host cell adsorption, including receptor binding protein (RBP), putative tail fiber proteins, encoded by ORF 22 and ORF 42, respectively, and other host-binding proteins, in SIA3lw. The SIA3lw RBP clusters with that of SPLA5b, suggesting SIA3lw may also use BtuB protein on the bacterial host for adsorption. However, phage SIA3lw could not infect *S.* Typhimurium as phage SPLA5b did through binding to the BtuB protein, vitamin B12 transport protein, on the bacterial outer membrane [30]. In contrast, phages SIA3lw and Bf23 can infect generic *E. coli* strains, which also harbor BtuB proteins, with the additional function of transporting colicins expressed by some *E. coli* strains [47]. Since SIA3lw does not infect *S.* Typhimurium, the phage may utilize a different receptor on *S.* Infantis for host recognition and binding. Additionally, many T5-like phages use Ltfs, usually composed of LtfA and LtfB, to facilitate binding to lipopolysaccharide (LPS) O antigen [48]. Moreover, LtfA forms a primary functional Ltf, and LtfB serves as an additional Ltf to recognize other host receptor proteins [31, 48]. In contrast to other T5-like reference phages containing both LtfA and LtfB, phage SIA3lw in this study has a single gene encoding an Ltf variant, annotated as putative tail fiber protein, like phages T5 and Bf23. The SIA3lw Ltf was categorized in the LtfA cluster, including T5 Ltf, but was unique compared to that of the reference phages used in this study, likely contributing to the adsorption of the receptors on *S.* Infantis strains. Besides, SIA3lw contains the genes coding for putative peptidase S74 (ORF 44) and band 7 domain-containing proteins (ORF 99), both not found in the Bf23 genome. A previous study found that putative peptidase S74 contained a conserved C-terminal domain that formed the distal part of tail fiber proteins to bind to the host receptors [49, 50]. However, the band-7 protein family is a diverse group of membrane-bound proteins, but their exact functions are unknown [51]. These findings likely account for the different host specificities to *Salmonella* serovars between SIA3lw (against *S.* Infantis) and Bf23 (against *S.* Typhimurium). Without detecting known virulence, lysogenic, or antibiotic-resistant genes, phage SIA3lw can be safely used as a potential antimicrobial agent. Nevertheless, the primary binding site of the SIA3lw RBP and the mechanism of its Ltf upon the infection of *S.* Infantis require further studies to confirm.

Regarding the biological characterization of the one-step growth curve as a fundamental growth factor, phage SIA3lw has a relatively long latent period compared to other *Salmonella* phages [52, 53]; however, its large burst size of 150 PFU/CFU against *S.* Infantis is advantageous as a biocontrol agent to produce considerably more phage progenies for the subsequent infection [54]. Phage stability is critical to ensure the active status of phages so that they can successfully target their bacterial host upon application under various environmental conditions. SIA3lw can withstand pH 4 to pH 11 for 24 h, showing slightly higher pH stability than a polyvalent phage previously isolated in our lab [29]. Regarding thermal susceptibility, phage SIA3lw is stable at temperatures, ranging from 30 °C to 50 °C, without any reduction but remains viable at a low level, reducing from 8 to 2 log PFU/mL, after thermal treatment of 65 °C for 24 h. Furthermore, the phage remains stable at 4 °C for at least 128 days. These biological characteristics of SIA3lw ensure sufficient antimicrobial activity when applied in most food-processing environments; however, the application in extreme environments may require enhancement of phage viability through encapsulation or other delivery methods to increase antimicrobial efficacy [52].

Unlike other polyvalent phages targeting different pathogenic bacterial species [29, 55], SIA3lw also infects generic *E. coli* (ATCC 13706 and ATCC 15597) with a highly productive infection, which can be beneficial in terms of scale-up phage production and potential commercialization [56]. Phage SIA3lw demonstrated strong lytic effects against most *S.* Infantis strains except for two strains used in the current study: one showed resistance, and the other resulted in inefficient phage production after phage infection. Based on the in vitro antimicrobial activity, phage SIA3lw with MOI = 1 did not cause any reduction of two antibiotic-resistant *S.* Infantis FSIS9799 and FSIS4897 until 6-h and 4-h time points, respectively. Using MOI = 10, the antimicrobial activity of SIA3lw contributed to a higher reduction of *S.* Infantis FSIS4897 than *S.* Infantis FSIS9799 during the first 8-h treatment. The MOI = 100 had the best antimicrobial effects, causing 4.7 and 4.5 log reduction of *S.* Infantis FSIS9799 and *S.* Infantis FSIS4897, respectively, compared to the control groups at the 8-h time point. Together with the findings from other studies [34, 57], a high MOI is preferable to yield potent antimicrobial activity via increased contact probability between phages and their target bacteria, even though the effect of LO may occur. However, phage SIA3lw with MOI = 100 did not cause LO on *S.* Infantis strains, indicating high cell wall stability of the *Salmonella* strains [58]. Furthermore, this study showed that SIA3lw-treated bacterial populations with MOI of 10 and 100 started to grow after 8 h throughout the remaining period of the phage treatment. A similar phenomenon was found in a previous study using a lytic phage against *S.* Infantis spiked in milk, and after a 2-log bacterial reduction was achieved [59]. However, the authors did not elucidate the reason. The current results showed that bacterial bounceback was primarily due to the occurrence of presumptive BIMs of *S.* Infantis, closely related to higher MOIs, regardless of bacterial strains used in this study (Table S3). At the same time, these *S.* Infantis presumptive BIMs were still sensitive to the infection of another T4-like phage Sa45lw, which targeted different receptors from those of SIA3lw. T4-like phages use LPS or outer membrane protein C (OmpC) for the adsorption, while T5-like phages utilize outer membrane transporter proteins, such as FhuA or BtuB, at the outer membrane to initiate infection [13]. Therefore, more and more studies have proposed the use of phage combination for bacterial control to circumvent phage resistance [60, 61]. Interestingly, the screening results in this study showed that some *S.* Infantis (FSIS9799 and FSIS4897) presumptive BIMs obtained after the treatment of SIA3lw became sensitive to streptomycin at certain dosages. Similarly, Fong et al. also observed an increasing susceptibility of *S.* Agona BIMs to tetracycline obtained after phage treatment [62]. Although the authors did not find the mutations of the genes associated with antibiotic-resistant traits in the bacterial chromosome, they suspected that the possible alterations in transcriptional activity may be the cause of the phenotypic change. Another study examined the response of phage-resistant *E. coli* to the treatment of various antibiotics [63]. The authors found that four phage-resistant *E. coli* strains they sequenced had a mutation affecting LPS structure and synthesis—a phage receptor and barrier to prevent the entry of hydrophobic molecules, such as antibiotics—and 1 of 4 LPS mutants exhibited the loss in antibiotic resistance across 4 of the 8 antibiotics tested. They also revealed that some LPS mutants maintained membrane permeability and prevented antibiotics entry into the cells because the mutations did not impact the core polysaccharide structure. The phenomenon likely accounts for a small portion of phage-resistant mutants with trade-offs between phage susceptibility and antibiotic resistance in the current study, even though the proteins associated with phage binding and the barrier to antibiotics were different. Together with the current study, these findings shed light on the interaction of lytic phages and their target bacterial hosts, particularly those with antibiotic resistance, during phage applications. Additionally, phage SIA3lw could be a good candidate for hurdle interventions to improve phage efficacy in reducing antibiotic-resistant strains and minimizing phage resistance development.

## Conclusions

Our results show that phage SIA3lw is effective against antibiotic-resistant *S.* Infantis strains in vitro. The polyvalent effect of the phage targeting generic *E. coli* renders hassle-free scale-up phage propagation upon commercialization of this phage. Although BIMs occur after phage treatment, all presumptive BIMs are susceptible to infection by a different phage, with few trading off their antibiotic-resistant capacities, primarily streptomycin, for phage resistance. The findings of this study inspire lytic phages as a promising antibiotic alternative to combat antibiotic-resistant *S.* Infantis.

## Supplementary Information

Supplementary Material 1.

## Data Availability

The genome sequence of *Salmonella* phage vB_SalS-SIA3lw was deposited at the National Center for Biotechnology Information (NCBI) database under GenBank accession number # PP094244.

## Abbreviations

Phage: Bacteriophage
ORF: Open reading frame
PFU: Plaque-forming units
CFU: Colony-forming units
TEM: Transmission electron microscopy
OD_600_: Optical density at 600 nm
MOI: Multiplicities of infection
BIMs: Bacteriophage-insensitive mutants
XLD: Xylose lysine deoxycholate
TSA: Tryptic soy agar
TAL: Thin agar layer
TSB: Trypic soy broth
LB: Lysogeny broth
VICTOR: Virus Classification and Tree Building Online Resource
ICTV: International Committee on Taxonomy of Virus
Ltf: L-shape tail fiber
RBP: Receptor binding protein
