# Supplementary material for: A Tequintavirus bacteriophage SIA3lw isolated from sewage water with antimicrobial potential against antibiotic-resistant Salmonella Infantis
Source: BMC Microbiol. 2025 Nov 21;25:806. doi: 10.1186/s12866-025-04423-4 (PMC12752171; doi:10.1186/s12866-025-04423-4)
Supplement: Supplementary file 1 — Supplementary Material 1. [file 12866_2025_4423_MOESM1_ESM.docx]

**A *Tequintavirus* Bacteriophage SIA3lw Isolated from Sewage Water with Antimicrobial Potential against Antibiotic-Resistant *Salmonella* Infantis**

Yen-Te Liao, Angela Voelker, Mackenna Chu, Yujie Zhang, Kan-Ju Ho, Leslie A. Harden, Alexandra Salvador, and Vivian C.H. Wu *

Produce Safety and Microbiology Research Unit, Department of Agriculture, Agricultural Research Service, Western Regional Research Center, Albany, California 94710, United States of America

* Corresponding author email: vivian.wu@usda.gov

**Supplementary Information:**

**Table S1. Bacterial strains, including *Escherichia coli*, *Escherichia albertii*, and *Salmonella enterica*, used in the current study for host range test, efficiency of plating and phage application.**

**Table S2. List of annotated ORFs with location and direction in SIA3lw genome.**

**Table S3. Confirmation of presumptive *S.* Infantis FSIS4897 and FSIS9799 BIMs after 24-h phage treatment using different MOIs.**

**Fig S1. Uncropped sodium dodecyl sulfate-polyacrylamide gel (SDS-PAGE) image for phage SIA3lw proteins.**

**Table S1. Bacterial strains, including *Escherichia coli*, *Escherichia albertii*, and *Salmonella enterica*, used in the current study for host range test, efficiency of plating and phage application.**

| **Ref. No.** | **Strain** | **Source^a^** | **Serogroup** | ***eaeA*** | ***stx_1_*** | ***stx_2_*** |
| --- | --- | --- | --- | --- | --- | --- |
| SJ2 | *E. coli* O26:H11 | NA | 26 | NA | + | + |
| RM10729 | *E. coli* O45:H- | cattle | 45 | - | + | - |
| RM10744 | *E. coli* O103:H- | cattle feces | 103 | + | + | - |
| RM11765 | *E. coli* O111:H- | water | 111 | + | + | - |
| RM8082 | *E. coli* O121:H- | cattle feces | 121 | - | + | - |
| RM13514 | *E. coli* O145:H28 | outbreak strain | 145 | + | - | + |
| ATCC 35150 | *E. coli* O157:H7 | water | O157 | + | + | + |
| ATCC 43888 | *E. coli* O157:H7 | ATCC | O157 | + | - | - |
| ATCC15597 | Generic *E. coli* | ATCC |  |  |  |  |
| ATCC 13706 | Generic *E. coli* | ATCC |  |  |  |  |
| TVS353 | non-pathogenic *E. coli* | environment |  |  |  |  |
| RM9973 | *E. albertii* | Crow | - | + | - | + |
| RM9974 | *E. albertii* | Crow | - | + | - | + |
| RM15113 | *E. albertii* | Oregon Junco | - | + | - | + |
| RM15115 | *E. albertii* | Nuthatch | - | + | - | + |
| RM10705 | *E. albertii* | Cow bird | - | + | - | + |
| ATCC 14028 | *Salmonella* Typhimurium | ATCC |  |  |  |  |
| ATCC BAA-3142 | *Salmonella* Typhimurium | ATCC |  |  |  |  |
| S1 | *Salmonella* Montevideo | NA |  |  |  |  |
| H1073 | *Salmonella* Newport | NA |  |  |  |  |
| 45955 | *Salmonella* Heidelberg | NA |  |  |  |  |
| PT-30 | *Salmonella* Enteritidis | NA |  |  |  |  |
| ATCC BAA-1675 | *Salmonella* Infantis | ATCC |  |  |  |  |
| RM2480 | *Salmonella* Infantis | NA |  |  |  |  |
| RM2481 | *Salmonella* Infantis | NA |  |  |  |  |
| RM19091 | *Salmonella* Infantis | NA |  |  |  |  |
| RM19096 | *Salmonella* Infantis | NA |  |  |  |  |
| FSIS9799 | *Salmonella* Infantis | raw chicken |  |  |  |  |
| FSIS9916 | *Salmonella* Infantis | raw chicken |  |  |  |  |
| FSIS4897 | *Salmonella* Infantis | raw chicken |  |  |  |  |
| FSIS4900 | *Salmonella* Infantis | raw chicken |  |  |  |  |
| FSIS4921 | *Salmonella* Infantis | comminuted beef |  |  |  |  |
| FSIS9851 | *Salmonella* Infantis | raw chicken |  |  |  |  |
| FSIS9861 | *Salmonella* Infantis | raw chicken |  |  |  |  |
| FSIS7821 | *Salmonella* Infantis | raw chicken |  |  |  |  |
| FSIS5221 | *Salmonella* Infantis | comminuted turkey |  |  |  |  |
| FSIS7823 | *Salmonella* Infantis | raw chicken |  |  |  |  |

^a^The source is where the strain was originally isolated from.

“-“ is the negative and “+” is positive of the PCR results.

NA means the information is not available.

**Table S2. List of annotated ORFs with location and direction in SIA3lw genome.**

| **ORF** | **Name** | **Function** | **Minimum** | **Maximum** | **Length** | **Direction** |
| --- | --- | --- | --- | --- | --- | --- |
| 1 | hypothetical protein CDS | na | 1032 | 1145 | 114 | reverse |
| 2 | hypothetical protein CDS | na | 1142 | 1345 | 204 | reverse |
| 3 | DNA-(apurinic or apyrimidinic site) endonuclease CDS | DNA & RNA metabolism | 1371 | 1715 | 345 | reverse |
| 4 | hypothetical protein CDS | na | 1717 | 1929 | 213 | reverse |
| 5 | integral membrane protein CDS | cell lysis | 1932 | 2081 | 150 | reverse |
| 6 | hypothetical protein CDS | na | 2131 | 2361 | 231 | reverse |
| 7 | hypothetical protein CDS | na | 2466 | 2960 | 495 | reverse |
| 8 | hypothetical protein CDS | na | 3026 | 4036 | 1011 | reverse |
| 9 | hypothetical protein CDS | na | 4898 | 5032 | 135 | forward |
| 10 | hypothetical protein CDS | na | 5183 | 5386 | 204 | forward |
| 11 | baseplate wedge protein CDS | Structure/ host recognition | 5617 | 5868 | 252 | forward |
| 12 | A2 protein CDS | Structure/host lysis | 5965 | 6372 | 408 | forward |
| 13 | A1 protein CDS | Structure/ virion production | 6429 | 6626 | 198 | forward |
| 14 | DNA transfer protein CDS | Structure/host lysis | 6724 | 8388 | 1665 | forward |
| 15 | hypothetical protein CDS | na | 8455 | 8607 | 153 | forward |
| 16 | hypothetical protein CDS | na | 8582 | 8980 | 399 | forward |
| 17 | 5'-deoxyribonucleotide monophosphatase (dmp) CDS | Host regulation | 9060 | 9794 | 735 | forward |
| 18 | tail assembly protein CDS | Structure | 10199 | 10393 | 195 | reverse |
| 19 | tail fiber protein CDS | Structure | 10390 | 10497 | 108 | reverse |
| 20 | hypothetical protein CDS | na | 10497 | 10625 | 129 | reverse |
| 21 | receptor-blocking protein CDS | Other | 10793 | 11059 | 267 | reverse |
| 22 | receptor-binding protein CDS | Structure | 11145 | 12902 | 1758 | forward |
| 23 | putative terminase small subunit CDS | Phage DNA packaging | 12913 | 13395 | 483 | forward |
| 24 | terminase large subunit CDS | Phage DNA packaging | 13395 | 14711 | 1317 | forward |
| 25 | nicking site-specific endonuclease CDS | DNA & RNA metabolism | 14826 | 15263 | 438 | forward |
| 26 | portal (connector) protein CDS | Structure | 15263 | 16480 | 1218 | forward |
| 27 | tail fibers protein CDS | Structure | 16477 | 16971 | 495 | forward |
| 28 | capsid and scaffold protein CDS | Structure | 16975 | 17607 | 633 | forward |
| 29 | major capsid protein CDS | Structure | 17625 | 19001 | 1377 | forward |
| 30 | head completion protein CDS | Structure | 19061 | 19573 | 513 | forward |
| 31 | tail completion protein CDS | Structure | 19573 | 20340 | 768 | forward |
| 32 | tail tube terminator protein CDS | Structure | 20344 | 20829 | 486 | forward |
| 33 | tail tube protein (N4) CDS (need to update) | Structure | 20867 | 22273 | 1407 | forward |
| 34 | tail fibers protein CDS | Structure | 22278 | 23180 | 903 | forward |
| 35 | tape measure chaperone CDS | Structure | 23173 | 23577 | 405 | forward |
| 36 | tape measure chaperone CDS | Structure | 23639 | 24007 | 369 | forward |
| 37 | tape measure protein CDS | Structure | 24091 | 27771 | 3681 | forward |
| 38 | distal tail protein CDS | Structure | 27881 | 28495 | 615 | forward |
| 39 | tail protein CDS | Structure | 28492 | 31341 | 2850 | forward |
| 40 | tail protein CDS | Structure | 31341 | 33398 | 2058 | forward |
| 41 | putative tail protein CDS | Structure | 33402 | 33824 | 423 | forward |
| 42 | putative tail fiber protein CDS | Structure | 33824 | 36172 | 2349 | forward |
| 43 | hypothetical protein CDS | na | 36169 | 36444 | 276 | forward |
| 44 | putative peptidase S74 domain-containing protein CDS | Structure | 36454 | 38196 | 1743 | forward |
| 45 | deoxyUTP pyrophosphatase CDS | DNA & RNA metabolism | 38256 | 38702 | 447 | reverse |
| 46 | flap endonuclease CDS | DNA & RNA metabolism | 38699 | 39574 | 876 | reverse |
| 47 | protein D14 CDS | DNA & RNA metabolism | 39574 | 40056 | 483 | reverse |
| 48 | recombination related exonuclease CDS | DNA & RNA metabolism | 40060 | 41898 | 1839 | reverse |
| 49 | recombinase CDS | DNA & RNA metabolism | 41879 | 42856 | 978 | reverse |
| 50 | D11 protein CDS | DNA & RNA metabolism | 42893 | 43666 | 774 | reverse |
| 51 | hypothetical protein CDS | na | 43659 | 43943 | 285 | reverse |
| 52 | DNA helicase CDS | DNA & RNA metabolism | 44164 | 45516 | 1353 | reverse |
| 53 | hypothetical protein CDS | na | 45513 | 45800 | 288 | reverse |
| 54 | DNA-directed DNA polymerase CDS | DNA & RNA metabolism | 46003 | 48480 | 2478 | reverse |
| 55 | hypothetical protein CDS | na | 48664 | 48993 | 330 | reverse |
| 56 | DNA replication primase CDS | DNA & RNA metabolism | 49020 | 49910 | 891 | reverse |
| 57 | putative replicative DNA helicase CDS | DNA & RNA metabolism | 49907 | 51430 | 1524 | reverse |
| 58 | D5 protein CDS | DNA & RNA metabolism | 51462 | 52229 | 768 | reverse |
| 59 | DNA ligase CDS | DNA & RNA metabolism | 52222 | 52998 | 777 | reverse |
| 60 | DNA ligase CDS | DNA & RNA metabolism | 53201 | 54172 | 972 | reverse |
| 61 | transcriptional coactivator p15 (PC4) C-terminaldomain-containing protein CDS | DNA & RNA metabolism | 54532 | 54840 | 309 | reverse |
| 62 | hypothetical protein CDS | na | 54891 | 55187 | 297 | reverse |
| 63 | D3 protein CDS | DNA & RNA metabolism | 55224 | 55634 | 411 | reverse |
| 64 | D2 protein CDS | DNA & RNA metabolism | 55997 | 56701 | 705 | reverse |
| 65 | hypothetical protein CDS | na | 56770 | 57003 | 234 | reverse |
| 66 | DNA primase C CDS | DNA & RNA metabolism | 56987 | 59854 | 2868 | reverse |
| 67 | tail tube protein CDS | Structure | 60396 | 60788 | 393 | reverse |
| 68 | hypothetical protein CDS | na | 60798 | 61226 | 429 | reverse |
| 69 | RNA repair protein CDS | DNA & RNA metabolism | 61229 | 61735 | 507 | reverse |
| 70 | SIR2 family NAD-dependent protein deacetylase CDS | DNA & RNA metabolism | 61826 | 62644 | 819 | reverse |
| 71 | hypothetical protein CDS | na | 62637 | 62858 | 222 | reverse |
| 72 | hypothetical protein CDS | na | 62827 | 63030 | 204 | reverse |
| 73 | hypothetical protein CDS | na | 63027 | 63239 | 213 | reverse |
| 74 | ribonucleotide reductase of class III (Anaerobic) large subunit CDS | DNA & RNA metabolism | 63339 | 65213 | 1875 | reverse |
| 75 | phosphate starvation-inducible protein CDS | Other (Host regulation) | 65566 | 66318 | 753 | forward |
| 76 | tail fibers protein CDS | Structure | 66320 | 66562 | 243 | forward |
| 77 | ribonucleoside-diphosphate reductase CDS | DNA & RNA metabolism | 66579 | 69014 | 2436 | forward |
| 78 | ribonucleoside-diphosphate reductase CDS | DNA & RNA metabolism | 69121 | 70266 | 1146 | forward |
| 79 | dihydrofolate reductase CDS | DNA & RNA metabolism | 70263 | 70796 | 534 | forward |
| 80 | putative thymidylate synthase CDS | DNA & RNA metabolism | 70796 | 71635 | 840 | forward |
| 81 | hypothetical protein CDS | na | 71728 | 71982 | 255 | forward |
| 82 | hypothetical protein CDS | na | 71982 | 72251 | 270 | forward |
| 83 | ribonuclease H CDS | DNA & RNA metabolism | 72251 | 72727 | 477 | forward |
| 84 | hypothetical protein CDS | na | 72805 | 73083 | 279 | forward |
| 85 | hypothetical protein CDS | na | 73167 | 73682 | 516 | forward |
| 86 | tail length tape-measure protein CDS | Structure | 73747 | 73962 | 216 | forward |
| 87 | metallopeptidase CDS | DNA & RNA metabolism | 74004 | 74240 | 237 | forward |
| 88 | putative metallopeptidase CDS | DNA & RNA metabolism | 74269 | 74970 | 702 | forward |
| 89 | hypothetical protein CDS | na | 75041 | 75223 | 183 | forward |
| 90 | tail fiber protein CDS | Structure | 75277 | 75915 | 639 | forward |
| 91 | cyclic-phosphate processing receiver domain-containing protein CDS | Other (Host regulation) | 76358 | 76675 | 318 | forward |
| 92 | cell wall hydrolase CDS | cell lysis | 76681 | 77130 | 450 | forward |
| 93 | hypothetical protein CDS | na | 77199 | 77369 | 171 | forward |
| 94 | recombination related exonuclease CDS | DNA & RNA metabolism | 77369 | 77812 | 444 | forward |
| 95 | tRNA-Arg | tRNA | 78662 | 78736 | 75 | forward |
| 96 | band 7 domain-containing protein CDS | Structure | 78771 | 79718 | 948 | forward |
| 97 | DNA primase CDS | DNA & RNA metabolism | 79816 | 80334 | 519 | forward |
| 98 | tRNA-Ser | tRNA | 80355 | 80443 | 89 | forward |
| 99 | tRNA-Met | tRNA | 80450 | 80527 | 78 | forward |
| 100 | hypothetical protein CDS | na | 80547 | 80735 | 189 | forward |
| 101 | tRNA-Leu | tRNA | 80743 | 80819 | 77 | forward |
| 102 | hypothetical protein CDS | na | 80835 | 81002 | 168 | forward |
| 103 | hypothetical protein CDS | na | 80995 | 81201 | 207 | forward |
| 104 | hypothetical protein CDS | na | 81292 | 81576 | 285 | forward |
| 105 | tRNA-Tyr | tRNA | 81705 | 81795 | 91 | forward |
| 106 | tRNA-Glu | tRNA | 81803 | 81879 | 77 | forward |
| 107 | tRNA-Trp | tRNA | 81888 | 81964 | 77 | forward |
| 108 | tRNA-Phe | tRNA | 81971 | 82045 | 75 | forward |
| 109 | homing endonuclease CDS | DNA & RNA metabolism | 82063 | 82332 | 270 | forward |
| 110 | hypothetical protein CDS | na | 82383 | 82601 | 219 | forward |
| 111 | tRNA-Cys | tRNA | 82780 | 82855 | 76 | forward |
| 112 | tRNA-Asn | tRNA | 82863 | 82945 | 83 | forward |
| 113 | Phage protein CDS | na | 83048 | 83233 | 186 | forward |
| 114 | tRNA-Asp | tRNA | 83243 | 83319 | 77 | forward |
| 115 | hypothetical protein CDS | na | 83346 | 83693 | 348 | forward |
| 116 | hypothetical protein CDS | na | 84147 | 84305 | 159 | forward |
| 117 | tRNA-Pro | tRNA | 84310 | 84387 | 78 | forward |
| 118 | tRNA-Met | tRNA | 84394 | 84471 | 78 | forward |
| 119 | hypothetical protein CDS | na | 84492 | 84659 | 168 | forward |
| 120 | tRNA-Lys | tRNA | 84661 | 84739 | 79 | forward |
| 121 | tRNA-Ala | tRNA | 85026 | 85099 | 74 | forward |
| 122 | tRNA-Leu | tRNA | 85568 | 85652 | 85 | forward |
| 123 | hypothetical protein CDS | na | 85778 | 86008 | 231 | forward |
| 124 | tRNA-His | tRNA | 86578 | 86654 | 77 | forward |
| 125 | hypothetical protein CDS | na | 86680 | 86874 | 195 | forward |
| 126 | tRNA-Gln | tRNA | 86888 | 86963 | 76 | forward |
| 127 | tRNA-Gln | tRNA | 86970 | 87045 | 76 | forward |
| 128 | tRNA-Gly | tRNA | 87053 | 87127 | 75 | forward |
| 129 | hypothetical protein CDS | na | 87142 | 87393 | 252 | forward |
| 130 | hypothetical protein CDS | na | 87386 | 87550 | 165 | forward |
| 131 | tRNA-Thr | tRNA | 87561 | 87635 | 75 | forward |
| 132 | hypothetical protein CDS | na | 87710 | 88000 | 291 | forward |
| 133 | tRNA-Ile | tRNA | 88016 | 88092 | 77 | forward |
| 134 | tRNA-Met | tRNA | 88188 | 88263 | 76 | forward |
| 135 | hypothetical protein CDS | na | 88301 | 88495 | 195 | forward |
| 136 | pyruvate formate-lyase CDS | Host regulation | 88538 | 88906 | 369 | forward |
| 137 | hypothetical protein CDS | na | 88906 | 89067 | 162 | forward |
| 138 | hypothetical protein CDS | na | 89195 | 89302 | 108 | forward |
| 139 | hypothetical protein CDS | na | 89424 | 89771 | 348 | forward |
| 140 | hypothetical protein CDS | na | 89848 | 90129 | 282 | forward |
| 141 | hypothetical protein CDS | na | 90122 | 90421 | 300 | forward |
| 142 | hypothetical protein CDS | na | 90414 | 90809 | 396 | forward |
| 143 | hypothetical protein CDS | na | 91080 | 91364 | 285 | forward |
| 144 | hypothetical protein CDS | na | 91475 | 91819 | 345 | forward |
| 145 | hypothetical protein CDS | na | 91974 | 92672 | 699 | forward |
| 146 | I-spanin CDS | cell lysis | 92629 | 93078 | 450 | forward |
| 147 | deoxynucleotide monophosphate kinase CDS | DNA & RNA metabolism | 93362 | 94114 | 753 | forward |
| 148 | ATP-dependent Clp protease proteolytic subunit CDS | other | 94127 | 94726 | 600 | forward |
| 149 | holin CDS | cell lysis | 94856 | 95539 | 684 | forward |
| 150 | endolysin CDS | cell lysis | 95536 | 95949 | 414 | forward |
| 151 | hypothetical protein CDS | na | 96027 | 96443 | 417 | forward |
| 152 | hypothetical protein CDS | na | 96519 | 96950 | 432 | forward |
| 153 | thioredoxin CDS | other | 96943 | 97233 | 291 | forward |
| 154 | hypothetical protein CDS | na | 97361 | 97738 | 378 | forward |
| 155 | hypothetical protein CDS | na | 97743 | 97988 | 246 | forward |
| 156 | serine/threonine protein phosphatase CDS | other | 97991 | 98854 | 864 | forward |
| 157 | hypothetical protein CDS | na | 98854 | 99141 | 288 | forward |
| 158 | putative serine/threonine protein phosphatase CDS | other | 99141 | 99731 | 591 | forward |
| 159 | hypothetical protein CDS | na | 99899 | 100330 | 432 | forward |
| 160 | hypothetical protein CDS | na | 100409 | 100660 | 252 | forward |
| 161 | hypothetical protein CDS | na | 100824 | 101105 | 282 | forward |
| 162 | hypothetical protein CDS | na | 101102 | 101347 | 246 | forward |
| 163 | hypothetical protein CDS | na | 101337 | 101663 | 327 | forward |
| 164 | hypothetical protein CDS | na | 101660 | 101806 | 147 | forward |
| 165 | hypothetical protein CDS | na | 101763 | 101963 | 201 | forward |
| 166 | hypothetical protein CDS | na | 101960 | 102421 | 462 | forward |
| 167 | capsid and scaffold protein CDS | Structure | 102369 | 102740 | 372 | forward |
| 168 | homing endonuclease CDS | DNA & RNA metabolism | 102797 | 103450 | 654 | forward |
| 169 | hypothetical protein CDS | na | 103721 | 103954 | 234 | forward |
| 170 | hypothetical protein CDS | na | 103954 | 104139 | 186 | forward |
| 171 | hypothetical protein CDS | na | 104139 | 104744 | 606 | forward |
| 172 | hypothetical protein CDS | na | 104744 | 105232 | 489 | forward |
| 173 | hypothetical protein CDS | na | 105229 | 105954 | 726 | forward |
| 174 | hypothetical protein CDS | na | 106080 | 106220 | 141 | forward |
| 175 | hypothetical protein CDS | na | 107657 | 107770 | 114 | reverse |
| 176 | hypothetical protein CDS | na | 107767 | 107970 | 204 | reverse |
| 177 | DNA-(apurinic or apyrimidinic site) endonuclease CDS | DNA & RNA metabolism | 107996 | 108340 | 345 | reverse |
| 178 | hypothetical protein CDS | na | 108342 | 108554 | 213 | reverse |
| 179 | integral membrane protein CDS | cell lysis | 108557 | 108706 | 150 | reverse |
| 180 | hypothetical protein CDS | na | 108756 | 108986 | 231 | reverse |
| 181 | hypothetical protein CDS | na | 109091 | 109585 | 495 | reverse |
| 182 | hypothetical protein CDS | na | 109651 | 110661 | 1011 | reverse |
| 183 | hypothetical protein CDS | na | 111523 | 111657 | 135 | forward |
| 184 | hypothetical protein CDS | na | 111808 | 112011 | 204 | forward |
| 185 | baseplate wedge protein CDS | Structure/ host recognition | 112242 | 112493 | 252 | forward |
| 186 | A2 protein CDS | Structure/host lysis | 112590 | 112997 | 408 | forward |
| 187 | A1 protein CDS | Structure/ virion production | 113054 | 113251 | 198 | forward |
| 188 | DNA transfer protein CDS | Structure/host lysis | 113349 | 115013 | 1665 | forward |
| 189 | hypothetical protein CDS | na | 115080 | 115232 | 153 | forward |
| 190 | hypothetical protein CDS | na | 115207 | 115605 | 399 | forward |
| 191 | 5'-deoxyribonucleotide monophosphatase (dmp) CDS | Host regulation | 115685 | 116419 | 735 | forward |

na means the function is not known.

**Table S3. Confirmation of presumptive *S.* Infantis FSIS4897 and FSIS9799 BIMs after 24-h phage treatment using different MOIs.**

| **Strain ID** | **MOI** | **Number of colonies tested** | **Presumptive BIM*** | |
| --- | --- | --- | --- | --- |
|  |  |  | **Number** | **Percentage (%)** |
| FSIS4897 | 1 | 30 | 4 | 13.3 |
|  | 10 | 30 | 18 | 60.0 |
|  | 100 | 30 | 20 | 66.7 |
| FSIS9799 | 1 | 30 | 0 | 0.0 |
|  | 10 | 30 | 0 | 0.0 |
|  | 100 | 30 | 27 | 90.0 |

*Presumptive BIMs are confirmed after the initial screening using a spectrophotometer and/or spot test and the subsequent spot test after three runs of streaking on TSA plates.


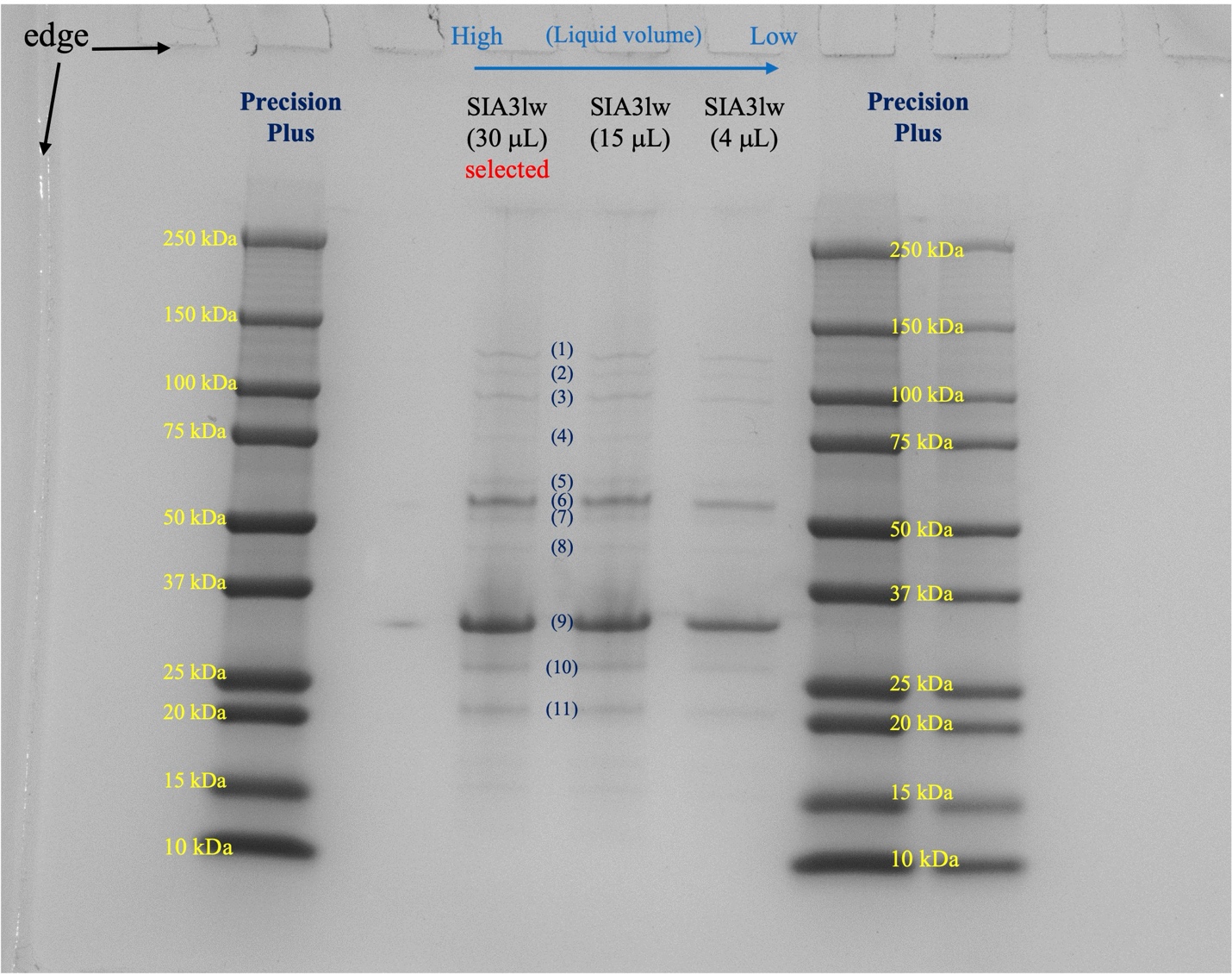


**Fig S1. Uncropped sodium dodecyl sulfate-polyacrylamide gel (SDS-PAGE) image for phage SIA3lw proteins.** The CsCl-purified phage SIA3lw was added with Laemmli buffer before loading 30 μL, 15 μL, and 4 μL into the wells from left to right of an SDS-PAGE. The lane loaded with 30 μL of the phage sample was used in Fig. 5 of the manuscript.
